# Influence of Feeding Type and *Nosema ceranae* Infection on the Gut Microbiota of *Apis cerana* Workers

**DOI:** 10.1128/mSystems.00177-18

**Published:** 2018-11-06

**Authors:** Shao K. Huang, Kun T. Ye, Wei F. Huang, Bi H. Ying, Xin Su, Li H. Lin, Jiang H. Li, Yan P. Chen, Ji L. Li, Xiu L. Bao, Jian Z. Hu

**Affiliations:** a College of Bee Science, Fujian Agriculture and Forestry University, Fujian, China; b USDA-ARS Bee Research Lab, Beltsville, Maryland, USA; c Key Laboratory of Pollinating Insect Biology of the Ministry of Agriculture, Institute of Apicultural Research, Chinese Academy of Agricultural Science, Beijing, China; d Department of Genetics and Genomic Sciences, Icahn School of Medicine at Mount Sinai, New York, New York, USA; Michigan State University

**Keywords:** *Apis cerana*, *Nosema ceranae*, food, gut, microbiota

## Abstract

The gut microbiota plays an essential role in the health of bees. To elucidate the effect of feed and Nosema ceranae infection on the gut microbiota of honey bee (Apis cerana), we used 16S rRNA sequencing to survey the gut microbiota of honey bee workers fed with sugar water or beebread and inoculated with or without N. ceranae. The gut microbiota of A. cerana is dominated by *Serratia*, *Snodgrassella*, and *Lactobacillus* genera. The overall gut microbiota diversity was show to be significantly differential by feeding type. N. ceranae infection significantly affects the gut microbiota only in bees fed with sugar water. Higher abundances of *Lactobacillus*, *Gluconacetobacter*, and *Snodgrassella* and lower abundances of *Serratia* were found in bees fed with beebread than in those fed with sugar water. N. ceranae infection led to a higher abundance of *Snodgrassella* and a lower abundance of *Serratia* in sugar-fed bees. Imputed bacterial Kyoto Encyclopedia of Genes and Genomes (KEGG) pathways showed the significant metagenomics functional differences by feeding and N. ceranae infections. Furthermore, A. cerana workers fed with sugar water showed lower N. ceranae spore loads but higher mortality than those fed with beebread. The cumulative mortality was strongly positive correlated (rho = 0.61) with the changes of overall microbiota dissimilarities by N. ceranae infection. Both feeding types and N. ceranae infection significantly affect the gut microbiota in A. cerana workers. Beebread not only provides better nutrition but also helps establish a more stable gut microbiota and therefore protects bees in response to N. ceranae infection.

**IMPORTANCE** The gut microbiota plays an essential role in the health of bees. Scientific evidence suggests that diet and infection can affect the gut microbiota and modulate the health of the gut; however, the interplay between those two factors and the bee gut microbiota is not well known. In this study, we used a high-throughput sequencing method to monitor the changes of gut microbiota associated with both feeding types and Nosema ceranae infection. Our results showed that the gut microbiota composition and diversity of Asian honey bee were significantly associated with both feeding types and the N. ceranae infection. More interestingly, bees fed with beebread showed higher microbiota stability and lower mortality rates than those fed with sugar water when infected by N. ceranae. Those data suggest that beebread has the potential not only to provide better nutrition but also help to establish a more stable gut microbiota to protect bees against N. ceranae infection.

## Abstract (precis)

The gut microbiota plays an essential role in the health of bees. Scientific evidence suggests that diet and infection can affect the gut microbiota and modulate the health of the gut; however, the interplay between those two factors and the bee gut microbiota is not well known. In this study, we used a high-throughput sequencing method to monitor the changes of gut microbiota associated with both feeding types and Nosema ceranae infection. Our results showed that the gut microbiota composition and diversity of Asian honey bee were significantly associated with both feeding types and the N. ceranae infection. More interestingly, bees fed with beebread showed higher microbiota stability and lower mortality rates than those fed with sugar water when infected by N. ceranae. Those data suggest that beebread has the potential not only to provide better nutrition but also help to establish a more stable gut microbiota to protect bees against N. ceranae infection.

## INTRODUCTION

European honey bees (Apis mellifera) and Asian honey bees (A. cerana) are two truly domesticated bee species that play a vital role in agriculture and ecosystem by providing pollination service to food crops and natural plants. However, both bee species are confronted with many biotic and abiotic stressors, including diseases caused by pathogens and parasites, acute and sublethal toxicity of pesticides, and malnutrition due to loss of foraging habitat; these stressors have acted separately or synergistically to cause significant declines of bee health and population worldwide (1–3). As a result, the health of managed honey bees has drawn much attention worldwide in recent years. There has been growing evidence that gut bacteria play very important roles in animal health by maintaining homeostasis, modulating immunity, regulating nutrition metabolism, and supporting host development and reproduction (4–6). The dynamics of gut microbial composition, diversity, and variability (stability) have been linked to the host response to diet (7), pathogens (8), chemical exposure (9), and other environmental stressors (10). Although most insect guts harbor relatively few microbiota species compared to mammalian guts, insect bacteria have been shown to be essential in regulating various aspects of their host biology (11–13). Over the past decade, progress has been made in understanding the composition and functional capacity of microbes living in honey bee guts (14–19). The honey bee gut microbiota is established gradually through trophallaxis, food consumption, and interactions with the hive environment (20). Many factors, such as genetics, age, diet, geography, and medication, can affect the composition of the gut microbiota (21–23). Several types of bacteria have been identified in the guts of A. mellifera, including the genera of *Bacillus*, *Lactobacilli*, and *Staphylococcus* from the *Firmicutes* phylum and *Coliforms* from the *Enterobacteriaceae* family of the *Proteobacteria* phylum (24–26).A previous study reported that species within the *Apis* genus share rather simple and similar gut bacterial microbiotas. Fewer than 10 members formed a core species, including *Lactobacillus*, *Bifidobacterium*, *Neisseria*, *Pasteurella*, *Gluconobacter* and the species of the newly named genera *Snodgrassella* and *Gilliamella* (27–29). However, both principal-coordinate analysis (PCoA) and nonmetric multidimensional scaling (NMDS) analysis of dissimilarities suggested that the overall gut communities of A. mellifera and A. cerana are clearly distinguishable (30), even in geographic locations where those two species co-occurred. Since most the studies about the microbiota in *Apis* were conducted in European honey bees (A. mellifera) and not in Asian honey bees (A. cerana), the influence of forms of environmental exposure such as diet and infection on the gut microbiota of A. cerana has barely been investigated.

Nosema ceranae is an intracellular parasitic microsporidium that disrupts the bee's digestive system. It was first identified in the A. cerana but has recently spread rapidly through A. mellifera bees (31–33). In A. mellifera, N. ceranae was found to seriously reduce the life expectancy of adults, decrease the productivity of the colony, and cause severe colony loss, especially during wintering in the temperate area (33–35). Furthermore, in A. mellifera, stresses caused by N. ceranae were shown to be more severe when mixed infections happened with other parasites or pathogens, such as *Varroa* mites and viruses (36–40). N. ceranae is now one of the major threats to the honey bee populations and has often been reported in honey bee colony losses worldwide (41, 42). A recent work has shown the correlation between diet-related gut bacterial dysbiosis in A. mellifera and the N. ceranae infection (7). Furthermore, eliminating gut flora by administration of antibiotics made A. mellifera more susceptible to N. ceranae infection (43). In consistency with the findings from studies of A. mellifera, a survey of microbial communities from the digestive tracts of A. cerana workers showed that N. ceranae infection might also have detrimental effects on the gut microbiota (1). However, the relationship between N. ceranae and microbiota in A. cerana is largely unknown. In this study, we challenged A. cerana workers with N. ceranae and then fed them with either beebread or sugar water. The intent of the current study was to evaluate the effects of N. ceranae infection and feed types on the gut microbiota.

## RESULTS

### Simple core bacterial clusters in the gut of Apis cerana.

**FIG 1:**
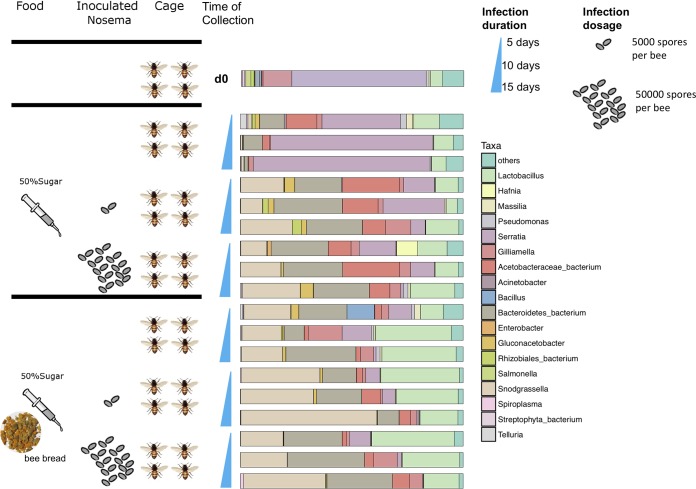
Experimental design and survey of the gut microbiota composition. Bar plots represent the mean relative abundances (%) of the gut microbiota of each group of bees fed with different feeding types and doses of N. ceranae infection at days 5, 10, and 15 (increasing order). The taxon classification is at the genus level.

As illustrated in Fig. 1, the A. cerana adult workers were grouped by feeding types and the level of N. ceranae infection. Analysis of the microbial composition of gut tissue collected at 5 days, 10 days, and 15 days postinfection (dpi) for each subgroup following a method described previously (26, 44) showed that the gut microbiota of A. cerana was rather simple and mainly contained three phyla, *Proteobacteria*, *Firmicutes*, and *Bacteroidetes*, accounting for over 97% of the total microbiota composition (see Fig. S1 in the supplemental material). At the genus level, fewer than 6 taxa from *Proteobacteria* and *Firmicutes* were dominant in the A. cerana gut bacterial community. In detail, they were the genera *Snodgrassella*, *Acetobacteraceae*, *Serratia*, *Gilliamella*, and *Lactobacillus* and unclassified genera from *Bacteroidetes*, among which *Serratia* was not in the core species clusters of A. mellifera (29).

10.1128/mSystems.00177-18.1 FIG S1 Variability of microbiota abundance across bees in each treatment and time point. Box plots show means and variances of results from three major phyla (*Proteobacteria*, *Firmicutes*, and *Bacteriodetes*) grouped by treatment (upper panel, sugar plus beebread; lower panel, sugar only) and time points (5 days, 10 days, and 15 days postinfection). Colors of the box plots indicate noninfected controls and low and high dosages of N. ceranae inoculations. Download FIG S1, PDF file, 0.04 MB. Copyright © 2018 Huang et al. 2018 Huang et al. This content is distributed under the terms of the Creative Commons Attribution 4.0 International license.

### Feeding type and N. ceranae infection changed the relative abundance of microbes in the gut.

**FIG 2:**
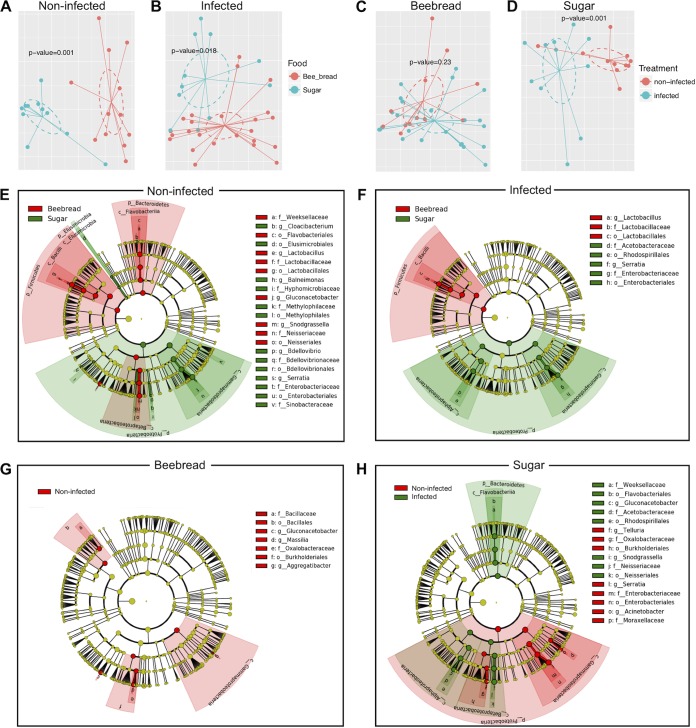
Feeding types and N. ceranae infection changed the relative abundances of microbes in the gut. Panels A to D show NMDS plots representing the overall dissimilarities between samples grouped by feeding types (A and B) or N. ceranae infection status (C and D). *P* values were given by PERMANOVA test. Panels E to H show cladogram plots as a visual way of representing significance and phylogeny. The colors represent which branch of the phylogenetic tree more significantly represents a certain group. In Panels E and F, red color indicates beebread-enriched taxa; green color indicates sugar-enriched taxa. In panels G and H, red color indicates enriched taxa in noninfected bees; green color indicates enriched taxa in infected bees. The brightness of each dot is proportional to its effect size.

The overall microbiota dissimilarity in samples grouped by feeding types or N. ceranae infection was visualized in NMDS plots (Fig. 2A to D). The overall gut microbiotas were significant different between bees fed with beebread and sugar (*P* = 0.018 with N. ceranae infection and *P* = 0.001 without infection [permutational multivariate analysis of variance {PERMANOVA} using Bray-Curtis distance]) (Fig. 2A and B). In sugar-fed bees, we found that N. ceranae infection significantly altered the microbiota (*P* = 0.001) (Fig. 2D). However, N. ceranae infection caused no significant alteration in gut microbiota in bees fed with beebread (*P* = 0.23) (Fig. 2C). The linear discriminant analysis effect size (LEfSe) algorithm was applied to select the microbiota taxa which are significantly associated with either feeding types or N. ceranae infections. In subgroups without N. ceranae infection (Fig. 2E and F), the bees fed with beebread (Fig. 2E) showed higher *Lactobacillus*, *Snodgrassella*, and *Weeksellaceae* abundance and less *Serratia* genus abundance than the bees fed with sugar. However, in the subgroups with N. ceranae infection (Fig. 2F), the bees fed with beebread showed more *Lactobacillus* abundance and less *Serratia* and *Acetobacteraceae* abundance than bees fed with sugar (Fig. 2). Among the bees fed with beebread (Fig. 2G), N. ceranae infection had a minor effect on microbiota, merely decreasing the abundance of the *Massilia*, *Aggregatibacter*, and *Gluconacetobacter* genera. Among bees fed with sugar solution (Fig. 2H), N. ceranae infection caused major changes in microbiota and was associated with increased *Weeksellaceae*, *Snodgrassella,* and *Gluconacetobacter* abundance and decreased abundance of *Proteobacteria* phyla, in particular, *Telluria*, *Serratia*, and *Acinetobacter*.

### Differential metagenome features predicted by PICRUSt and their association with feeding types and N. ceranae infection status.

We performed PICRUSt (phylogenetic investigation of communities by reconstruction of unobserved states) analysis to predict the full metagenomic content of microbial communities using 16S gene surveys (45) and compared the predicted metagenomic pathways by feeding types and N. ceranae infection status (Fig. S1). The nearest sequenced taxon index (NSTI), which quantifies the uncertainty of the prediction (lower values mean a better prediction), ranged from 0.027 to 0.11 with a mean value of 0.067, indicating fair reliability and accuracy in the metagenome reconstruction. The heat map (Fig. S2) with clustering analysis showed the overall changes in predicted Kyoto Encyclopedia of Genes and Genomes (KEGG) pathways. Among the significantly differential pathways, we found that the feeding types could affect bacterial glycolysis/gluconeogenesis, fructose and mannose metabolism, and metabolism of several amino acids. N. ceranae infection could affect biosynthesis of several amino acids, the signal transduction mechanism, and the lipopolysaccharide biosynthesis and phosphotransferase system (PTS).

10.1128/mSystems.00177-18.2 FIG S2 Heat map of metagenome features predicted by PICRUSt and their association with feeding types and N. ceranae infection. The heat map was created using the R package *ComplexHeatmap*. Each column corresponds to a specific sample and each row to a KEGG pathway predicted by PICRUSt. The proportions that each lineage contributed to the full population within each sample are indicated with the color scale to the right of the figure (values from −2 to 2). Metadata are color-coded at the top and include feeding types and N. ceranae infection. The KEGG pathways were clustered using average linkage hierarchical clustering as the default and split by kmeans = 5. The mean abundances of the pathways ranged from 0% to 6% and are shown with gradual color changes. A nonparametric Wilcoxon test with false-discovery-rate (FDR)-adjusted *P* values was performed for analysis of feeding types (pvalue1) or infection (pvalue2). The labeled KEGG pathways are those with >0.5% mean abundance and that showed significance (*P* < 0.05) for either feeding types (green color) or infection (red color). Download FIG S2, PDF file, 0.4 MB. Copyright © 2018 Huang et al. 2018 Huang et al. This content is distributed under the terms of the Creative Commons Attribution 4.0 International license.

### The cumulative mortality of caged bees with different feeding type and infection status.

**FIG 3:**
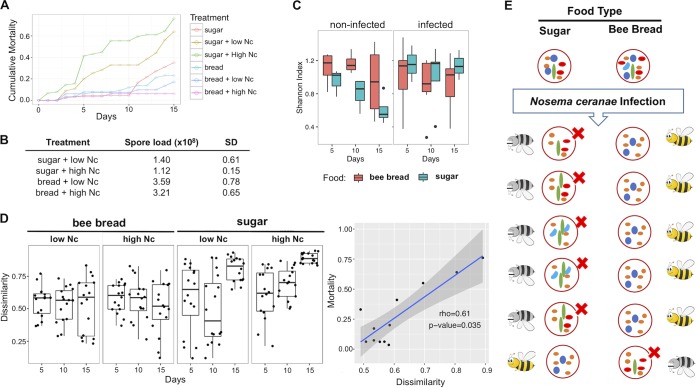
Differences in bee mortalities and stabilities of the gut microbiota resulting from feeding and N. ceranae infections. (A) Cumulative mortalities under different treatment conditions. (B) Means and standard deviations of the N. ceranae (Nc) spore load under different treatment conditions. (C) Alpha diversity of the midgut microbiota under different treatment conditions. (D) Means and variances of the dissimilarities (beta diversity) of the midgut microbiota under different treatment conditions. (E) Illustration of the links between the decreased stability in midgut microbiota and the increased bee mortality in sugar-fed bees.

When caged bees were inoculated with N. ceranae spores, the average cumulative mortality increased gradually during the experimental observation (Fig. 3A). Compared to those fed with beebread, bees fed with only sugar water showed significantly shortened longevity after infection with N. ceranae (Fig. 3A). Interestingly, the spore loads in the gut of bees fed with beebread were significantly higher than in those fed with sugar water (*P* = 0.01 and 0.007 for low N. ceranae spore loads and high N. ceranae spore loads, respectively) at 15 days after inoculation (Fig. 3B). This was consistent with an earlier report by Zheng et al. (46). There was no interaction between feeding types and spore dosage with respect to mortality (*P* =0.868, *F* = 0.029). There was no significant difference in gut N. ceranae spore counts between low- and high-dosage N. ceranae inoculations. This may have been due to the late sampling time point with respect to the time at which the spore load in the gut had reached a plateau.

### The richness of the gut microbiota of caged bees with different feeding type and infection status.

We used the Shannon index, a commonly used metric, for assessment of richness within a given community (47). Without N. ceranae infection, the richness of the microbiota in bees fed with sugar solution was significantly lower than in those fed with beebread (Fig. 3C) (*P* < 0.05 at 5, 10, and 15 days). Furthermore, the richness of the microbiota decreased over time in bees fed with sugar but not in bees fed with beebread. In subgroups with N. ceranae infection, the microbiota of bees fed with sugar showed increased richness at all time points (with a slightly higher mean) but no significant differences from that of bee fed with beebread.

### The stability of the gut microbiota is significantly correlated with the cumulative mortality.

To define the stability of a microbial community under a given treatment condition, we measured the microbial β-diversity sample to sample variability using the Bray-Curtis dissimilarity index “D” parameter. Higher variability (higher D) between samples within a given group indicates less stability of microbial community in the group. The stability of the microbiota in response to N. ceranae infection in groups maintained under different feeding conditions showed that the bees fed with beebread showed relatively stable microbiota. The mean levels of dissimilarity were not significantly different for either sampling time postinfection or N. ceranae doses. However, among bees fed with sugar solution, the microbiota dissimilarities significantly increased by time, with the greatest dissimilarities and higher consistency seen at 15 days with the highest N. ceranae infection (Fig. 3D), suggesting the most divergent microbiota within this group. Further, the mean microbiota dissimilarities were significantly correlated with the cumulative mortality rate (*r* = 0.61, Spearman correlations, *P* =0.035).

## DISCUSSION

Our results demonstrated that the gut microbiotas of the A. cerana adult workers are composed of three major phyla, *Proteobacteria*, *Firmicutes*, and *Bacteriodetes*. This result is consistent with previous reports (28) except that the most abundant taxon in our study was *Proteobacteria*, which was the second most abundant taxon in the study by Ahn et al. (28). At the genus level, we found that the gut microbiota of Asian honey bees is dominated by a few core bacterial species. *Lactobacillus*, *Snodgrassella*, and *Gilliamella* were among the major genera found in both our study and previous studies (48).

Food constituents can influence the gut microbiota composition. Our results confirmed that feeding types significantly shape the composition of the gut microbiota of bees (Fig. 1 and 2) and metagenomic functions (see Fig. S2 in the supplemental material). Beebread contains high levels of protein and comprehensive nutrients, which may favor those proteolytic bacterial species in gut microflora. Furthermore, beebread also contains additional microbiotas (49, 50), in particular, *Lactobacillus*, *Bifidobacterium*, and other lactic acid bacteria, which may benefit the gut microflora (51).

Analysis of inferred metagenomes indicated that the altered gut microbiome was adapting the changes in feeding sugar levels through a number of metabolic pathways, in particular, the bacterial pathways associated with glycolysis/gluconeogenesis and fructose and mannose metabolism. It is also interesting that the N. ceranae infection led to differential bacterial lipopolysaccharide biosynthesis results, suggesting a possible role of lipopolysaccharide-mediated inflammation in changes in the physiology of the bees. Thus, the link between gut microbiota, dysregulated metabolic pathways, and the health of bees should be evaluated further in detail.

Our study also showed that the *Lactobacillus* and *Snodgrassella* genera were much more abundant in those bees fed with beebread (Fig. 2B). Members of the genera *Lactobacillus* and *Bifidobacterium* and the family *Pasteurellaceae* were also found in beebread from colonies of A. mellifera (46). *Lactobacillus* had been found in flora and hive environments, including honey, royal jelly, and beebread. *Lactobacillus* bacteria were also found in honey crops and showed an inhibitory effect on Paenibacillus larvae *in vitro* (52). It is likely that the *Lactobacillus* bacteria found in the gut of adult workers fed with beebread were obtained through food trophallaxis as previously described (53). In contrast, bees fed only with sugar showed greater levels of abundance of the *Serratia* genus of *Enterobacteriaceae* family. The presence of *Serratia* was further suggested to likely represent S. marcescens by sequencing nearly full-length 16S rRNA genes (data not shown), although additional analysis will be needed to confirm this. S. marcescens is commonly found in adult A. mellifera and A. cerana and in bumble bee gut. It is in general commensal with respect to honey bee and has been used to examine the host immune reaction to microbes (54). However, S. marcescens can also be pathogenic to bees and had been reported to cause detrimental effects on A. mellifera survivorship after erasure of host microbiota by antibiotics (55). Overgrowth of species of the *Enterobacteriaceae* family has been linked to gut inflammation in many studies in human and rodents (56, 57); however, the pathogenic potential of those species in bees is often underestimated as the cause of bee mortality (58, 59). In our study, sugar-fed bees with enriched *Enterobacteriaceae* (*Serratia*) in the gut microflora were likely more susceptible to the N. ceranae infection and showed a high overall mortality rate. Future investigations are necessary to further explore the underlying role of those opportunistic pathogens in bee guts.

N. ceranae resides in bee guts, and infection by N. ceranae can profoundly change the physiology of honey bees (60) and the host-microbiota relationship in the gut. An investigation conducted by Li et al. showed that four common bacterial clusters, *Bifidobacterium*, *Neisseriaceae*, *Pasteurellaceae*, and *Lactobacillus*, were less abundant in N. ceranae-infected adult A. cerana workers than in noninfected ones (1). However, we found minor changes in gut microbiota caused by N. ceranae infection in beebread-fed bees. The differences between the studies may have been a result of the use of the V3-V4 region of the 16S rRNA gene and of next-generation sequencing (NGS) in our study rather than the classical PCR and cloning methods as described previously (1). When sugar water was the only food supplied, N. ceranae infection strongly impacted the overall gut microbiota, with increased abundance of *Neisseriaceae/Snodgrassella*, *Weeksellaceae*, and *Gluconacetobacter* and decreased abundance of *Serratia*, *Telluria*, and *Enterobacteriaceae*. We found that N. ceranae infection caused much higher cumulative mortality in bees fed with sugar than in bees fed with beebread. Interestingly, the microbiome variability (stability) was highly correlated to the mortality of the bees. N. ceranae infection caused significant increases in both microbiota richness and dissimilarity in sugar-fed bees but not in beebread-fed bees. We speculated that N. ceranae infection combined with sugar feeding resulted in a more divergent microbiota in bee populations and that, among those with various gut flora statuses, the majority are dysbiotic and disease prone (Fig. 3E). In contrast, the gut microbiota in bees fed with beebread showed much lower sample-to-sample variability with N. ceranae infection. We speculated that beebread feeding may cause the levels of the members of the gut microbiota to converge into a more stable and symbiotic status which could play a protective role against N. ceranae infection and result in lower levels of mortality.

Our results suggest that the gut dysbiosis/symbiosis condition may affect the efficacy of N. ceranae infection. This finding helps us to understand the controversial results reported from earlier studies, in which sugar water-fed bees with lower N. ceranae spore loads showed higher mortality than pollen-fed bees with higher N. ceranae spore loads (46).

In summary, the gut microbiotas of A. cerana workers are significantly differentiated by both feeding type and N. ceranae infection. The higher stability of the gut microbiota in the bees fed with beebread plays a role in the ability of bees to resist N. ceranae infection and warrants further exploration.

## MATERIALS AND METHODS

### Honey bees.

Three A. cerana colonies without identified diseases were chosen for sample collection. Those colonies are located at the campus of College of Bee Science, Fujian Agriculture and Forestry University, Fuzhou, Fujian, China. Capped brood combs with pupae near emergence were taken from the colonies and then kept in an incubator at 35 ± 1°C and 55% to 65% relative humidity (RH). Workers that emerged within 24 h were collected for the study.

### Purification of Nosema ceranae spore.

Because the prevalence and spore loads of N. ceranae in A. cerana are lower than in A. mellifera (61, 62), N. ceranae spores were purified from A. mellifera foragers (62, 63). First, adult workers were captured at entrances of A. mellifera colonies and immobilized in a refrigerator for few minutes. The guts of the bees were then dissected, pooled, and ground in a mortar. Afterward, the suspension was purified by differential centrifugation (three runs at 300 × *g* for 3 min and 1,000 × *g* for 10 min alternately) to exclude most of the debris; finally, the suspension was loaded on Percoll (Sigma-Aldrich, St. Louis, USA) and centrifuged at 11,300 × *g* for 35 min (Sigma 3K15 centrifuge; China) to eliminate unsaturated spores (61). After centrifugation, the mature spores were extracted and washed three times with sterile ultrapure water. The purity and maturity of spore were further confirmed under phase-contrast microscopy. The spore species was confirmed as solely N. ceranae by an N. ceranae-specific PCR method (64). After counting was performed by the use of a hemacytometer, the spore was diluted to 10^9^ spores/ml of water and kept in water at 4°C for usage within 1 week.

### Treatments and sampling.

The newly emerged (<24 h) workers were randomly distributed into 18 laboratory rearing cages. A total of 30 bees were transferred to each cage. The experimental cages were divided into two groups. The first group was given 50% (wt/vol) sugar water without beebread in a modified syringe feeder (65), and the second group was given both 50% (wt/vol) sugar water and beebread freshly collected from the A. cerana colonies (here called “beebread”). For each group, three subgroups were set up based on the N. ceranae spore inoculation. One subgroup without spore inoculation was served as the negative control, one subgroup was inoculated with N. ceranae at 5,000 spores per bee, and one subgroup was inoculated with 50,000 spores per bee (Fig. 1). Each subgroup was maintained in three cages as replicates. The cages were kept in an incubator at 30 ± 1°C and 55% to 65% RH. Feed was changed each other day; dead bees were counted and removed every day. Eight or nine bee workers were collected per treatment and time point at days 5, 10, and 15 posttreatment. The gut tissue was individually collected sequentially from each bee at 5, 10, and 15 dpi and then stored in a freezer at −80°C until the subsequent microbial composition analysis.

### DNA extraction from gut tissue samples.

Sample bees were taken out of the refrigerator and rinsed with 7% benzalkonium bromide for 2 min and then rinsed four times with sterilized water to minimize the bacterial contamination from the body surface. The intestine tissues were collected with tweezers, clamping the end of the abdomen, and each gut tissue was further separated and transferred into a labeled 1.5-ml tube on ice. The entire procedure was conducted under aseptic conditions, and all tools used were sterilized. The total DNA of the gut tissue samples was extracted using Insect DNA Extraction Kit II (Beijing Demeter Biotech Ltd., Beijing, China), following the manufacturer’s instruction. The quality and yield of DNA samples were assessed using a Quawell Q5000 ultraviolet light-visible light (UV-Vis) spectrophotometer (Quawell, San Jose, CA, USA). DNA samples which did not meet the quality control sequencing requirement were discarded; finally, in total, 76 DNA samples from bee workers (12 samples per treatment group × 6 treatment groups plus 4 samples from the bees at day 0) were used for further analysis.

### Gut Nosema ceranae spore counting.

After caged bees were sampled at days 5, 10, and 15 posttreatment, the quantity of the spores in the gut specimen was counted as previously described (61) with slight modifications. Briefly, the sediments of gut were resuspended in 100 μl double-distilled water (ddH_2_O) and then subjected to evenly performed vortex mixing. The suspension was loaded onto the hemocytometer for N. ceranae spore inspection and counting under a microscope. We conducted three to four repeated measurements for each sample.

### Bacterial 16S rRNA gene PCR amplification.

The phylogenetically informative V3-V4 region of the 16S rRNA (rRNA) gene was amplified using universal primer 347F/803R (66, 67). The dual-barcoding approach was applied as previously described (68, 69) to label the 16S rRNA gene amplicons of each sample. Briefly, the 6-mer barcodes were attached on the 5′ ends of both forward and reverse PCR primers such that the 16S rRNA gene PCR amplicons from each sample contained a unique dual-barcode combination. The PCR primers were synthesized by Sangon Biotech, Shanghai, China, and the primer sequences are shown in Table S1 in the supplemental material. The 25-μl PCR mixture contained 300 ng of sample DNA as a PCR template, 1 μl of 10 μM forward and reverse 16S primers, and 12.5 μl of 2× HotMaster *Taq* DNA mix (Tiangen Biotech, Beijing, China). The PCR was performed on an Applied Biosystems model 2720 thermal cycler (Thermo Fisher Scientific Inc., Waltham, MA, USA) at 94°C for 3 min and then at 94°C for 30 s, 58°C for 30 s, and 72°C for 20 s for 30 cycles followed by 72°C for 4 min. The integrity of the PCR products was verified by agarose gel electrophoresis. After a purification step was performed with a gel purification kit (Promega, Madison, WI, USA), the 16S PCR amplicons were pooled at equal levels of molarity, freeze-dried, and submitted to the New York Genome Center for sequencing.

10.1128/mSystems.00177-18.3 TABLE S1 PCR primers for bacterial 16S sequencing. Download Table S1, DOCX file, 0.01 MB. Copyright © 2018 Huang et al. 2018 Huang et al. This content is distributed under the terms of the Creative Commons Attribution 4.0 International license.

### 16S rRNA gene sequencing and microbiota profiling.

The 16S rRNA gene PCR amplicons were sequenced on an Illumina HiSeq platform using 2 × 250 paired-end fast-run mode. In total, we generated 21 million high-quality 16S reads obtained by NGS on pooled barcoded PCR amplicons from 76 samples. After splitting by barcodes was performed, ∼ 2.5 × 10^5^ reads per sample were obtained. After the merge, the sequencing reads with a length of >400 and a quality score of >Q30 at more than 99% of bases were further split by barcode and trimmed of primer regions using CLC Genomic workbench 6 (Qiagen Bioinformatics, Redwood City, CA, USA). The filtered and trimmed high-quality reads were further processed by the use of QIIME 1.9.0 (70). We used the command *pick_open_reference_otus.py* with the default cutoff of 97% for a cluster of nearly identical sequencing reads as an operational taxonomic unit (OTU) using *Uclust* (71). Representative sequences for each OTU were aligned using PyNAST. Finally, the program built a biom-formatted OTU table with assigned taxonomical information for each of the OTUs. Using Chimera Slayer (72), chimera sequences arising from the PCR amplification were detected and excluded from the aligned representative sequences and the OTU table.

### Statistical analysis.

Mortality data from the different groups were transformed by square root and degrees and Asin and were then compared by using two-way analysis of variance (ANOVA; SPSS program). The overall microbiota dissimilarities among all samples were assessed using Bray-Curtis distance matrices (73) generated at the genus level. To test the significance of the overall microbiota differences between the gut microbiota grouped by feeding types and N. ceranae infections, the PERMANOVA (permutational multivariate analysis of variance) procedure (74, 75) was performed using the (Adonis) function of the *R* package *vegan* 2.0-5, with the maximum number of permutations = 999. The diversity within each microbial community (the so-called alpha-diversity) was calculated using the Shannon index as the metric, and the results represented the measure of the diversity at the genus level (47). Using the linear discriminant analysis (LDA) effect size (LEfSe) method (76), we further selected the microbiota features significantly associated with feeding types and N. ceranae infections. The program PICRUSt (phylogenetic investigation of communities by reconstruction of unobserved states) (45) was used to predict the metagenome functional content based on our 16S rRNA gene sequencing data. Briefly, a close reference-based OTU table was generated using the QIIME pipeline and input into PICRUSt to bin individual bacterial genes into Kyoto Encyclopedia of Genes and Genomes (KEGG) pathways, thereby predicting their function.

### Data availability.

16S rRNA gene sequencing information has been deposited in the European Nucleotide Archive with study accession number PRJEB21090.
