# Supplementary figures and images for: Influence of Feeding Type and Nosema ceranae Infection on the Gut Microbiota of Apis cerana Workers
Source: mSystems. 2018 Nov 6;3(6):e00177-18. doi: 10.1128/mSystems.00177-18 (PMC6222045; doi:10.1128/mSystems.00177-18)

# Sugar + Beebread

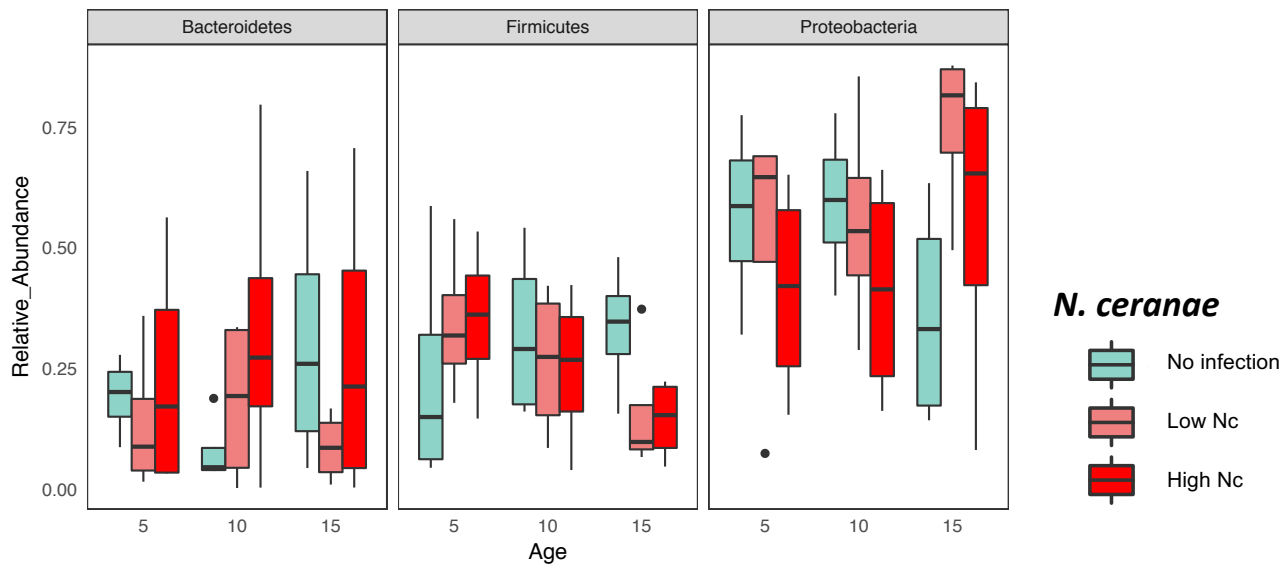

# Sugar

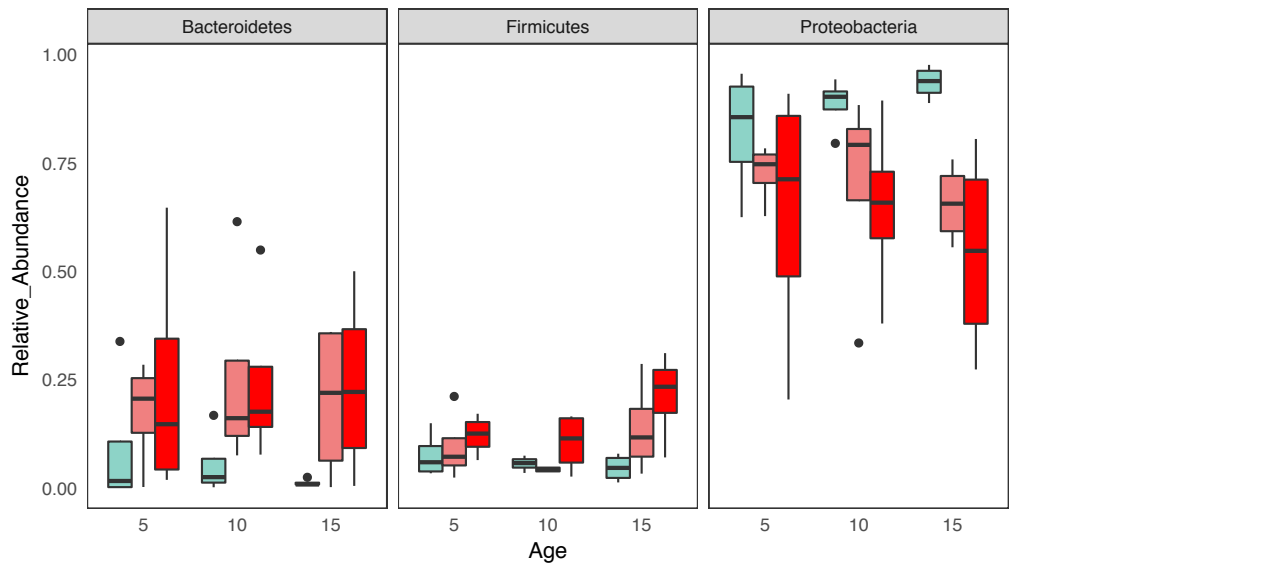

Supplement: FIG S1 [file sys006182287sf1.pdf]

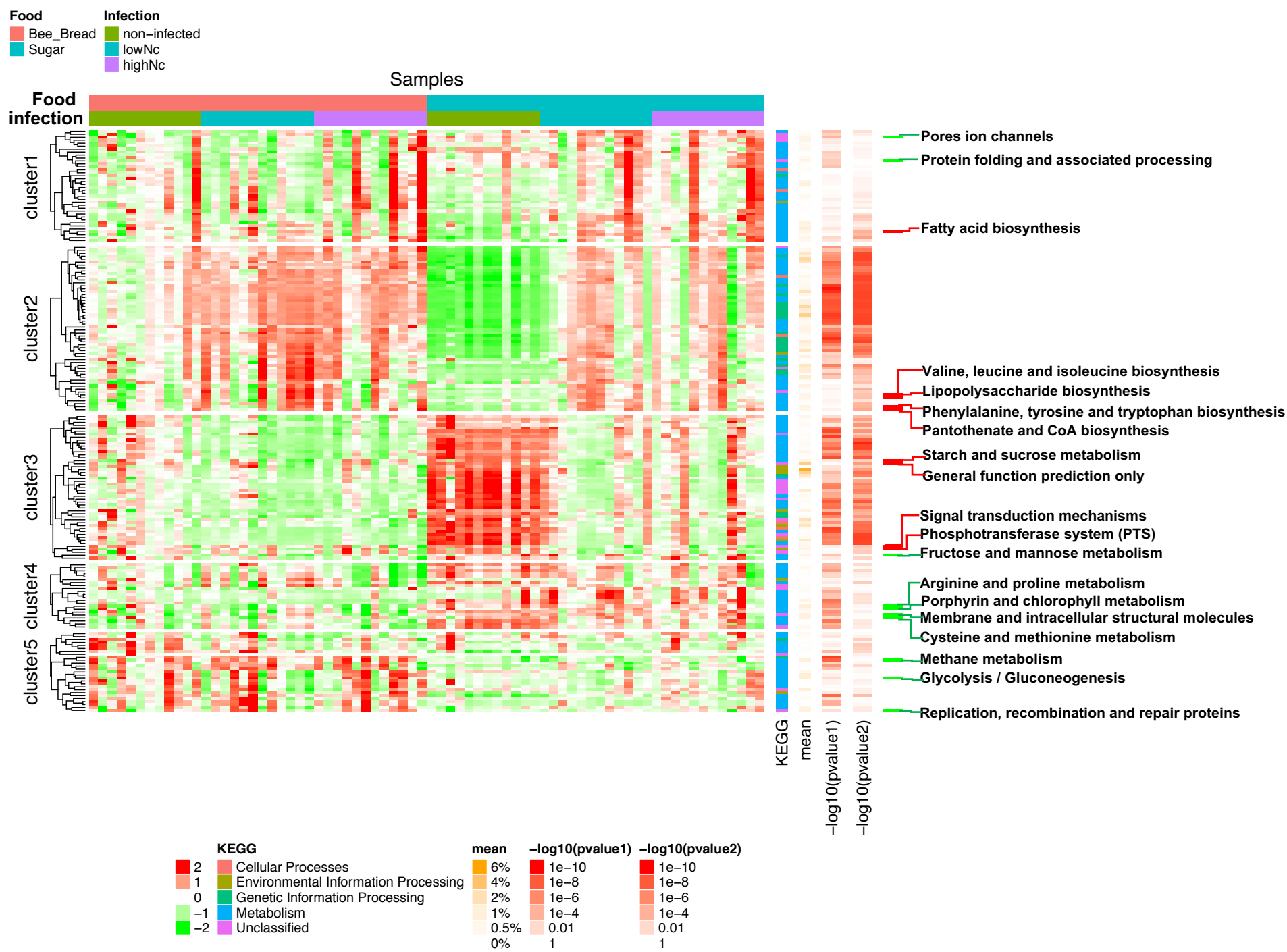

Supplement: FIG S2 [file sys006182287sf2.pdf]
