# Supplementary material for: Influence of Feeding Type and Nosema ceranae Infection on the Gut Microbiota of Apis cerana Workers
Source: mSystems. 2018 Nov 6;3(6):e00177-18. doi: 10.1128/mSystems.00177-18 (PMC6222045; doi:10.1128/mSystems.00177-18)
Supplement: TABLE S1 [file sys006182287st1.docx]

**Supplementary Table 1.** PCR primers for bacterial 16S sequencing

| **Primer Name** | **Sequence** |
| --- | --- |
| **16SF1** | AGTCGACAGGAGGCAGCAGTRRGGAAT |
| **16SF2** | AGGAACTCGGAGGCAGCAGTRRGGAAT |
| **16SF3** | AGCTGTAGGGAGGCAGCAGTRRGGAAT |
| **16SF4** | AGGACACGGGAGGCAGCAGTRRGGAAT |
| **16SF5** | AGAGCGAGGGAGGCAGCAGTRRGGAAT |
| **16SF6** | AGTCTCTAGGAGGCAGCAGTRRGGAAT |
| **16SF7** | AGCGTGTCGGAGGCAGCAGTRRGGAAT |
| **16SF8** | AGATGCGTGGAGGCAGCAGTRRGGAAT |
| **16SF9** | AGAACGCAGGAGGCAGCAGTRRGGAAT |
| **16SF10** | AGATTACCGGAGGCAGCAGTRRGGAAT |
| **16SF11** | AGTGGTCAGGAGGCAGCAGTRRGGAAT |
| **16SF12** | AGCCGTTTGGAGGCAGCAGTRRGGAAT |
| **16SR1** | AGTCGACACTACCRGGGTATCTAATCC |
| **16SR2** | AGGAACTCCTACCRGGGTATCTAATCC |
| **16SR3** | AGCTGTAGCTACCRGGGTATCTAATCC |
| **16SR4** | AGGACACGCTACCRGGGTATCTAATCC |
| **16SR5** | AGAGCGAGCTACCRGGGTATCTAATCC |
| **16SR6** | AGTCTCTACTACCRGGGTATCTAATCC |
| **16SR7** | AGCGTGTCCTACCRGGGTATCTAATCC |
| **16SR8** | AGATGCGTCTACCRGGGTATCTAATCC |
| **16SR9** | AGAACGCACTACCRGGGTATCTAATCC |
| **16SR10** | AGATTACCCTACCRGGGTATCTAATCC |
| **16SR11** | AGTGGTCACTACCRGGGTATCTAATCC |
| **16SR12** | AGCCGTTTCTACCRGGGTATCTAATCC |
